# Relationship of work-family conflict with burnout and marital satisfaction: cross-domain or source attribution relations?

**DOI:** 10.15171/hpp.2016.05

**Published:** 2016-03-31

**Authors:** Razieh Bagherzadeh, Ziba Taghizadeh, Eesa Mohammadi, Anoshirvan Kazemnejad, Abolghasem Pourreza, Abbas Ebadi

**Affiliations:** ^1^Department of Midwifery and Reproductive Health, Tehran University of Medical Sciences, Tehran, Iran; ^2^Department of Nursing, Tarbiat Modares University, Tehran, Iran; ^3^Department of Biostatistics, Tarbiat Modares University, Tehran, Iran; ^4^Department of Health Management and Economics, Tehran University of Medical Sciences, Tehran, Iran; ^5^Behavioral Sciences Research Center (BSRC), Baqiyatallah University of Medical Sciences, Tehran, Iran

**Keywords:** Burnout, Marital satisfaction, Work-family conflict

## Abstract

**Background:** The present study was conducted to examine the relationship between two dimensions of work-family conflict (WFC) with marital satisfaction and burnout in a society in which few studies have been done about the consequences of WFC.

**Methods:** A cross-sectional study was conducted in 2015. Surveys were distributed to 420 employed married women with various jobs living in Bushehr province, Iran. Data were collected using a questionnaire for demographic characteristic, the Netmeyer’s WFC questionnaire, Maslach Burnout Inventory: General Survey (MBI-GS), and Enrich maritalsatisfaction questionnaire. The data were analyzed using descriptive and inferential statistics.

**Results:** There was a negatively significant association between work interference with family(WIF) and overall burnout as well as emotional exhaustion (P < .01). Family interference with work (FIW) was significantly associated with depersonalization (P < .01). The overall marital satisfaction and its subscales were significantly associated with WIF (P < .01) and FIW (P < .01 for overall marital satisfaction and P < .05 for its subscales).

**Conclusion:** In terms of practical implication, to avoid creating disadvantages of WIF and FIW,facilitation in two domains of improving work and family conditions can be a useful means to prevent WFC and its consequences.

## Introduction

 Similar to the western countries, socio-demographic changes is occurring in the work force in the Iranian society.^1^ Recent evidence suggest that compared to the other parts of the world, women’s participation in the work force is limited in the Middle Eastern countries, including Iran. However, it seems that this trend is increasing so that it was increased from 14% to 18% from 2000 to 2014.^2^

 With increasing trend in the participation of women in the work force and due to the competing demands between work and family, the metaphor of work-family conflict (WFC) as an increasing pressure in professional life has emerged.^3,4^ WFC seems to be more common in women than men due to more overload, stress, and work-family conflicts.^5^

 WFC can lead to negative consequences in both work and family domain. A better understanding of this problem is an important step in helping families in preventing negative family-related consequences of the WFC. Organizations can also benefit from the research to be conducted in this area.^6^

 The majority of studies on WFC have been conducted in developed countries so far.^7^ Very few studies have been done in Iran about the consequences of WFC.^8,9^ There is also an increasing understanding regarding the impact of culture on WFC. It is therefore important to understand this issue better to help employees to handle the negative consequences of WFC.^6^

## Work-family conflict and its relationship with marital satisfaction and burnout

 WFC can be defined as: “the degree to which participation in one role inter­feres with one’s ability to meet the responsibilities of the other role.”^10^ In the past, this construct was assumed to be one-dimensional by many researchers; then, it has been shown that WFC is bi-directional. This means that the conflict arises when work-related roles interfere with family-related roles (WIF) and family-related roles interfere with work-related roles (FIW).^11-13^

 There are two perspective models about the relationship between two dimensions of WFC and its outcomes. Domain specificity (cross-domain) perspective suggests that WIF predominantly influences the family, while FIW affects the work. This is based on the assumption that the conflict in each domain can cause problems in the other domain.^12,14-16^

 The negative consequences of WFC can be divided into 3 categories: (1) work-related, (2) family-related, and (3) domain- unspecific outcomes.^15^ Marital satisfaction and burnout are two important outcomes of WFC in work and family domains, respectively. Burnout is a common problem in the work force.^17^ Blanch and Aluja found the relationships between WIF and burnout.^18^ Peeters et al conducted a cross-sectional study and showed that work-home interference and home-work interference had a mediating role in the relationship between job as well as home demands and burnout.^19^ A study on Australian cancer workers showed that burnout could mediate the relationship between WFC and intention to leave the organization.^20^ In the study by Wang et al on Chinese doctors, both WIF and FIW were positively associated with emotional exhaustion and depersonalization (two dimensions of burnout). FIW was negatively related to professional efficacy (another dimension of burnout).^21^ A cross-sectional study by Sholi et al on the employees of a gas company showed a positive relationship between WFC and burnout.^9^ Farhadi et al demonstrated an association between WFC, burnout, and intention to leave among female nurses.^8^

 Another consequence of WFC is decreased marital satisfaction. Several previous studies have examined the association between WFC and marital satisfaction and found mixed results. Judge et al found that WFC conflict was negatively associated with marital satisfaction.^22^ The study by Perrone et al showed that lower WFC was related to higher marital satisfaction.^23^ Eftekharsoadi and Bavi represented a negative relationship between FIW and marital satisfaction. However, they did not find any relationship between WIF and marital satisfaction.^24^

## Rationale for the study

 There were two reasons for conducting this study: First, to examine the relationship of two dimensions of WFC with marital satisfaction and burnout in a society, in which few studies have been done about the consequences of WFC; and second, to test whether the relationship between WIF and FIW with burnout and marital satisfaction adjusted to source attribution perspective. The research hypotheses were formulated as follows:

 Hypothesis 1: WIF and FIW are associated with burnout and its subscales.

 Hypothesis 2: WIF and FIW are associated with marital satisfaction and its subscales.

 Hypothesis 3: Relationship between WIF and burnout is stronger than its relationship with marital satisfaction and the relationship between FIW and marital satisfaction is stronger than its relationship with burnout (attribution perspective).

## Materials and Methods

###  Participants and Procedures 

 A cross-sectional study was conducted in 2015. Surveys were distributed to 420 employed married women with various jobs living in Bushehr province, Iran. Sampling method was clustering. Among the cities of Bushehr province, 4 cities were randomly selected from different parts of the province. Samples were selected from private and governmental job centers of these cities.

 Only participants who were members of dual career family, were married, living with their husbands, and working outdoors were included in the study.

 Questionnaires were delivered by the researchers to each individual, group, or working section and collected by the same researchers during a visit after 3 days to one week to the individuals, groups, and work sections. The purpose of this research was explained for all the participants and they made voluntary contribution to the study. Four hundred twelve surveys were collected. Twelve questionnaires were eliminated because of the large missing data and, finally, the analysis was conducted on 400 samples (response rate 95%).

###  Measures

 Demographic and job-related information including age, education (1=Illiterate and primary school; 2=Pre-high school; 3=High school diploma; 4=Associate’s degree; 5=Bachelor’s degree; and 6=Master’s or doctoral degrees), occupational status (1=Worker; 2=Officer; and 3= Self-employed), economic status (1=Good; 2=Moderate; and 3=Bad), work shift (1=Fixed; and 2=Not fixed), number of children, job tenure and working hours were recorded.

 Two subscales of the Persian version of Maslach Burnout Inventory: General Survey (MBI-GS) were used to assess burnout: Emotional exhaustion with 9 items measured feelings of being emotionally drained and exhausted by one’s work and depersonalization with 5 items measured an impersonal response to the recipients of one’s services, care, or instruction. These two dimensions are generally taken into account as the core of burnout, whereas professional efficacy (the third dimension of Maslach Burnout Inventory) reflects a personality characteristic rather than a genuine burnout component.^25^ All the items were scored on a 7-point rating scale which ranged from 0 (*never*) to 6 (*daily*). Higher scores indicated higher levels of burnout. The steps of translation and psychometric evaluation of this questionnaire have been previously conducted in Iran.^26^ In the current research, alpha reliabilities for emotional exhaustion, depersonalization and total scale were .862, .851 and .90 respectively.

 Marital satisfaction was evaluated by Enrich marital satisfaction questionnaire, which consists of 35 items and 4 subscales including ideal distortion (5 items), marital satisfaction (10 items), communication (10 items), and conflict resolution (10 items). All the items were answered on a 5-point scale from *strongly disagree* to *strongly agree*; higher scores indicated higher levels of marital satisfaction. The steps of translation and psychometric evaluation of this questionnaire have been previously conducted in Iran.^27^ In the current research, alpha reliabilities for ideal distortion, marital satisfaction, communication, conflict resolution, and overall scale were .802, .801, .822, .70, and .928, respectively.

 WFC was measured using a questionnaire obtained from Netmeyer et al work.^28^ This questionnaire includes two subscales: The first subscale contains 5 items which asks about the influence of work on personal/family life (WIF). The second subscale has 5 items assessing the interference of family with work (FIW). Both subscales were answered on a 5-point scale ranging from *strongly disagree* to *strongly agree*, with higher scores indicating higher levels of WFC. The English language of this questionnaire was translated into Persian and, then, back translated into English. Content validity was confirmed by using subject matter experts. In the current research, alpha reliabilities for WIF, FIW, and total scale were .872, .861, and .896, respectively.

###  Statistical analysis

 Cronbach alpha coefficients were used to evaluate the reliability of the constructs. Descriptive statistics (including means and standard deviations) and inferential statistics were used to analyze the data.

 Pearson product-momentum correlation coefficients were used to determine the relationships between the dependent and independent variables.

 Also, to predict the relationship between predictor variables and outcome, hierarchical regressions were used. Among the demographic variables, only age, work hours, and education were considered as the control variable (to avoid multicollinearity). Overall burnout and its subscales, and overall marital satisfaction and its subscales were regressed on age, work hours, and education in the first step as the control variable. In the second step, WIF and FIW (as predictor variable) were added. The level of statistical significance was set at *P*<.05.

## Results

 Means and standard deviations or frequency and percentage among the demographic variables are shown in Table 1. Intercorrelations coefficients (Pearson correlation test), means, and standard deviations of the dependent and independent variables are shown in Table 2. For hypothesis testing hierarchical regression analysis was used. Results of regression analysis are presented in Tables 3 and 4.

**Table 1:** Study participants’ characteristics

**Variable**	
Age of subjects (years) (mean±SD)	35.07±7.62
Marriage Lengths (years) (mean±SD)	11.48±8.29
Number of children (mean±SD)	1.45±1.13
Job tenure (years) (mean±SD)	10.63±7.42
Educational level of subjects (n=399), n (%)	
Pre-high school	20 (5)
Diploma	87 (21.8)
Associate’s degree	62 (15.5)
Bachelor’s degree	198 (42.5)
Master’s or doctoral degree	32 (8)
Occupational status of subjects (n=396), n (%)	
Worker	19 (4.8)
Officer	276 (69.7)
Self-employed	101(25.5)
Shift work (n=399), n (%)	
Fixed	250 (62.7)
No fixed	149 (37.3)
Economic status (n=400), n (%)	
Good	202 (50.5)
Moderate	147 (36.7)
Bad	51(12.8)

**Table 2:** Means, standard deviations and Intercorrelations among the dependent and independent variable

	**Mean**	**SD**	**1**	**2**	**3**	**4**	**5**	**6**	**7**	**8**	**9**	**10**	**11**
Age	35.07	7.62	1										
Work hours	44.11	17.67	-.149^**^	1									
WIF	15.81	4.70	.006	.173^**^	1								
FIW	13.02	4.43	-.043	.089	.578^**^	1							
Ideal distortion	17.19	4.24	-.225^**^	.083	-.226^**^	-.225^**^	1						
Communication	33.13	7.34	-.107^*^	-.028	-.301^**^	-.292^**^	.691^**^	1					
Conflict resolution	30.65	5.72	-.109^*^	.006	-.208^**^	-.200^**^	.523^**^	.752^**^	1				
Marital satisfaction	34.94	6.57	-.142^*^	-.029	-.378^**^	-.307^**^	.775^**^	.780^**^	.596^**^	1			
Emotional exhaustion	12.85	11.65	-.085	.090	.503^**^	.345^**^	-.147^**^	-.308^**^	-.202^**^	-.304^**^	1		
Depersonalization	4.03	4.73	-.130^*^	.116^*^	.164^**^	.217^**^	-.169^**^	-.240^**^	-.104	-.251^**^	.429^**^	1	
Burnout	32.82	18.72	-.143*	.108	.412**	.328**	-.270**	-.371**	-.211**	-.384**	.794**	.638**	1
Overall marital satisfaction	115.86	20.92	-.186**	-.002	-.304**	-.314**	.820**	.938**	.842**	.903**	-.272**	-.128	-.351**

Note: *P<.05, **P<.01.

**Table 3:** Regression analyses for WIF/ FIW and subscale of marital satisfaction and burnout

**Variables**	**Subscales of marital satisfaction**								**Subscales of burnout**			
	**Marital satisfaction**		**Communication**		**Conflict resolution**		**Ideal distortion**		**Emotional exhaustion**		**Depersonalization**	
	Step1	Step 2	Step 1	Step 2	Step 1	Step 2	Step 1	Step 2	Step 1	Step 2	Step 1	Step 2
Age	-.153*	-.170**	-.069	-.078	-.092	-.098	-.199**	-.199**	-.092	-.111*	-.133*	-.129*
Work hours	-.043	.035	-.015	.045	.012	.059	.072	.120*	.051	-.059	.114	.083
Education	.072	.069	.178**	.162**	.236**	.231**	.077	.066	-.053	-.027	-.002	.027
WIF		-.316**		-.247**		-.178**		-.182**		.497**		.087
FIW		-.171*		-.168*		-.133*		-.145*		.090		.193**
R^2^	.028*	.210**	.037*	.168**	.062**	.135**	.052**	.133**	.016	.308	.035*	.097**
R^2^ Change		.182**		132**		.073**		.081**		.291**		.062**

Note: *P<.05, **P<.01.

**Table 4:** Regression analyses for WIF/ FIW and overall marital satisfaction and burnout

**Variables**	**Overall Marital Satisfaction**		**Burnout**	
	Step 1	Step 2	Step 1	Step 2
Age	-.153*	-.178**	-.120*	-.139**
Work hours	.000	.075	.051	-.040
Education	.184**	.170**	-.066	-.019
WIF		-.249**		.432**
FIW		-.211**		.123
R^2^	.059**	.213**	.025	.275**
R^2^ Change		.154**		.250**

Note: *P<.05, **P<.01.

 The first hypothesis was concerned with whether WIF was associated with the overall burnout, emotional exhaustion, and depersonalization at the same point in time when controlling for other variables. There was a significantly negative association between WIF and overall burnout as well as emotional exhaustion (*P*<.01), but not between WIF and depersonalization (*P*>.05). FIW was significantly associated with depersonalization (*P*<.01), but there was no significant association between FIW and overall burnout as well as emotional exhaustion (*P*>.05). These findings partially supported the first hypothesis.

 The overall marital satisfaction and its subscales were significantly associated with WIF (*P*<.01). FIW was significantly associated with overall marital satisfaction (*P*<.01) as well as its subscales (*P*<.05), which supported the second hypothesis. The association between WIF and burnout was stronger than its relationship with marital satisfaction. On the other hand, the relationship between FIW and marital satisfaction was stronger than its relationship to burnout, which supported the third hypothesis.

## Discussion

 The present study had three main goals: (1) To examine whether WIF and FIW were related to overall burnout and its subscales; (2) To examine the relationship between WIF/FIW and overall marital satisfaction and its subscales; and (3) To examine whether the relationship between two dimensions of WFC and its outcome adjusted with source attribution perspective.

 The results indicated that the studied control variables accounted for little variance; also, WIF and FIW accounted for maximum variance in burnout and overall marital satisfaction. Burnout and emotional exhaustion were positively associated with WIF, but their association with FIW was not significant. The association between depersonalization and WIF was not significant, but there was a negatively significant relationship between depersonalization and FIW.

 In some studies, WFC was regarded as a whole and the bidirectional nature of this concept was not considered. Some of these studies have demonstrated a significant relationship between WFC, emotional exhaustion, and depersonalization^8,9,29^; which was generally in agreement with the finding of the present study, but ignoring the bidirectional nature of WFC could make the interpretation and comparison of these result completely difficult. In the study by Blanch and Aluja, WIF but not FIW was associated with burnout, which was consistent with the present work.^18^ In the meta-analysis conducted by Amstad ei al,^15^ burnout/exhaustion was associated with WIF and FIW. The result of this study about the relationship between FIW and burnout/exhaustion was not consistent with those of the present study. This inconsistency can be related to the combination of burnout and exhaustion as an outcome of WIF and FIW.

 Koekemoer and Mostert showed that negative work-home interaction was positively related to emotional exhaustion, but not related to depersonalization.^30^These findings were consistent with the present finding about the association between WIF, emotional exhaustion, and depersonalization. In this study, negative home-work interaction was not considered. In the investigation by Peeters et al,^19^ work-home interference was moderately correlated with exhaustion and weakly with depersonalization, which was consistent with the finding by the present study. Presumably, the complex nature of WFC, research population, considering subscales of latent variables, applied questionnaire, and statistical methods are responsible for the controversy of the research findings.

 The finding about the relationship between subscales of burnout with WIF and FIW was explained in terms of women’s attitude about work and family in Iran and the nature of questions which measured the dimension of job burnout. Most of the Iranian women believe housekeeping is the main responsibility of women and their responsibility to their family is a priority for them, so when their job interferes with their family, they are bored with their jobs and emotional exhaustion occurs. They relate their exhaustion to their job, because household tasks are the primary responsibility for women and every woman can afford to do it. On the other hand, when family interferes with job, they do not try to resolve it by decreasing the share of housework. As a result, depersonalization occurs, which is the feelings of frustration, hopelessness, disillusionment, and distrust regarding economic or governmental organizations, managers, and/or other aspects of job.

 Marital satisfaction and its subscales were negatively related to WIF and FIW. Some studies have represented a negative and significant relationship between WFC as a one-factor construct and marital satisfaction.^23,31,32^ Most studies have considered two dimensions of WFC, but they have regarded marital satisfaction as the overall construct and have not examined its subscale. Results of these studies are mixed. Some of these studies have demonstrated a negative relationship between two dimensions of WFC and marital satisfaction, which is consistent with the present study.^15,22^ Eftekharsoadi and Bavi showed a negative relationship between FIW and marital satisfaction. However, they did not find any relationship between WIF and marital satisfaction.^24^ These findings disagreed with the present study about the association between WIF and marital satisfaction. Such inconsistency might be due to research population. In the study by Eftekharsoadi and Bavi, the research subjects were male and female employees of Islamic Azad University, Ahwaz Branch, but in the present study, the samples were chosen from among the people with various jobs. Negative relationship between two dimensions of WFC and marital satisfaction suggested that marital satisfaction might play a mediating role between WIF and FIW. WIF decreased marital satisfaction, which would lead to FIW.

 WIF had a stronger relationship with job burnout (as a work-related outcome) than marital satisfaction (as a family-related outcome). On the other hand, FIW had a stronger relationship with marital satisfaction than burnout, which confirmed the source attribution relationship between two dimensions of WFC and its consequences, and was consistent with the study by Amstad et al,^15^ and Shockley and Singal.^16^ These findings provide further evidence that WIF is more caused by work than by family and stressors. Moreover, WIF has its consequences mainly in the work, than in family and environments.^33^ Likewise, FIW seems to be created mainly by family than by work stressors, and has its consequences mainly in the family than in the work domain. The source attribution relationship shows complexity of WFC phenomenon. It seems the interaction of the work and family domain has a spiral rather than unidirectional nature. For example, burnout (as a work stressor) and marital satisfaction (as a family stressor) may be act as the antecedents for WIF and FIW, respectively. Studies have shown that WIF and FIW have stronger relationships with job and family stressors, respectively.^12,14^

 In terms of practical implication, to avoid creating disadvantages of WIF and FIW, facilitation in two domains of work improvement is important to prevent WIF and its consequences such as burnout and promote marital satisfaction. To improve marital satisfaction, it is important to decrease FIW and WIF. To minimize FIW, governmental and organizational supports seem to be beneficial. Moreover, parents are needed to be prepared with effective parenting/communication skills to appropriately deal with the family challenges.^34^

## Limitations

 The present study had some limitations that should be taken into account. First, the cross-sectional nature of these data precluded the possibility to establish the directionality of causal relationships between two dimensions of WFC with marital satisfaction and burnout. Additional research of a longitudinal/or quasi-experimental nature is needed to further validate the hypothesized causality of the relationships. Second, the sample was limited to married, working women living in a certain province of Iran. However, unlike most studies which use one organization or one occupation, the sample of the present study represented a wide variety of occupations. Third, the data in this study were obtained using self-report measures, which may lead to data contamination due to common method variance.

## Ethical approval

 The protocol of study was approved by Ethical Committee of the Tehran University of Medical Sciences. Participation of people in study was completely voluntary. The purpose of the study was described to all the participants and they were assured that their personal information would remain confidential. Then, their consent letter for participation in the research was obtained.

## Competing interests

 The authors declare that there is no conflict of interests.

## Acknowledgments

 Authors acknowledge all participants involved in the project. We would like to thank Tehran University of medical sciences for financial support.

## References

[1] Karimi L, Nouri A (2009). Do work demands and resources predict work-to-family conflict and
facilitation? A study of Iranian male employees. J Fam Econ Issues.

[2] The World Bank. World Development Indicators: Labor force structure. [Internet]. The World Bank; 2015 [updated 2016 Feb 17; cited 2016 March 7]; Available from: http://wdi.worldbank.org/table/2.2#.

[3] Eby LT, Casper WJ, Lockwood A, Bordeaux C, Brinley A (2005). Work and family research in IO/OB: Content analysis and review of the
literature (1980–2002). J Vocat Behav.

[4] Martín-Fernández S, de Los Rios I, Cazorla A, Martínez-Falero E (2009). Pilot study on the influence of stress caused by the need to combine work
and family on occupational accidents in working women. Saf Sci.

[5] Van Daalen G, Willemsen TM, Sanders K (2006). Reducing work–family conflict through different sources of social
support. J Vocat Behav.

[6] Esson PL. Consequences of work-family conflict: Testing a new model of work-related, non-work related and stress-related outcomes [Master Thesis]. Virginia: Polytechnic Institute and State University; 2004.

[7] Aycan Z. Cross-cultural approaches to work-family conflict. In: Korabik K, Lero D, editors. Handbook of Work-Family Integration: Research, Theory and Best Practices. London: Cambridge University Press; 2008. p. 353-70.

[8] Farhadi A, Movahedi Y, Nalchi M, Daraei M, Mohammadzadegan R (2013). The relationship between Work-family conflict, burnout dimensions and
intention to leave among female nurses. Iran Journal of Nursing.

[9] Sholi S, Beshlideh K, Hashemi Sheykhshabani S, Arshadi N (2011). An investigation of the relationship between neuroticism, work-family
conflict, role overload, procedural justice, distributive justiceand job control with
job burnout in employees of Ahvaz gas company. Journal of Psychological Achievement.

[10] Greenhaus JH, Beutell NJ (1985). Sources of conflict between work and family roles. Acad Manage Rev.

[11] Carlson DS, Kacmar KM (2000). Work–family conflict in the organization: do life role values make a
difference?. J Manage.

[12] Frone MR, Yardley JK, Markel KS (1997). Developing and testing an integrative model of the work–family
interface. J Vocat Behav.

[13] Hashim N, Ishar NI, Rashid WE, Masodi MS (2012). Personality traits, work-family conflict and job satisfaction: items
validity using rasch measurement approach. Procedia Soc Behav Sci.

[14] Frone MR, Russell M, Cooper ML (1992). Antecedents and outcomes of work-family conflict: testing a model of the
work-family interface. J Appl Psychol.

[15] Amstad FT, Meier LL, Fasel U, Elfering A, Semmer NK (2011). A meta-analysis of work–family conflict and various outcomes with a special
emphasis on cross-domain versus matching-domain relations. J Occup Health Psychol.

[16] Shockley KM, Singla N (2011). Reconsidering work–family interactions and satisfaction: A
meta-analysis. J Manage.

[17] Maslach C, Jackson SE (1981). The measurement of experienced burnout. J Occup Behav.

[18] Blanch A, Aluja A (2012). Social support (family and supervisor), work–family conflict, and burnout:
Sex differences. Hum Relat.

[19] Peeters MC, Montgomery AJ, Bakker AB, Schaufeli WB (2005). Balancing Work and Home: How Job and Home Demands Are Related to
Burnout. Int J Stress Manag.

[20] Rani Thanacoody P, Bartram T, Casimir G (2009). The effects of burnout and supervisory social support on the relationship
between work-family conflict and intention to leave: astudy of Australian cancer
workers. J Health Organ Manag.

[21] Wang Y, Liu L, Wang J, Wang L. Work-family conflict and burnout among Chinese doctors: the mediating role of psychological capital. J Occup Health 2012;54:232-240. 10.1539/joh.11-0243-OA. 22790526

[22] Judge TA, Ilies R, Scott BA (2006). Work–family conflict and emotions: Effects at work and at
home. Pers Psychol.

[23] Perrone KM, Webb LK, Blalock RH (2005). The effects of role congruence and role conflict on work, marital, and life
satisfaction. J Career Dev.

[24] Eftekharsoadi Z, Bavi S (2012). Relationships between Work- Family conflict and family-work conflict with
job engagement and marital satisfaction. J Soc Psychol.

[25] Montgomery AJ, Panagopolou E, de Wildt M, Meenks E (2006). Work-family interference, emotional labor and burnout. Journal of Managerial Psychology.

[26] Yousefy A, Ghassemi GR (2006). Job burnout in psychiatric and medical nurses in Isfahan, Islamic Republic
of Iran. East Mediterr Health J.

[27] Asoodeh MH, Daneshpour M, Khalili S, Lavasani MG, Shabani MA, Dadras I (2011). Iranian successful family functioning: Communication. Procedia Soc Behav Sci.

[28] Netemeyer RG, Boles JS, McMurrian R (1996). Development and validation of work–family conflict and family–work conflict
scales. J Appl Psychol.

[29] Noor NM, Zainuddin M (2011). Emotional labor and burnout among female teachers: work–family conflict as
mediator. Asian J Soc Psychol.

[30] Koekemoer FE, Mostert K (2006). Job characteristics, burnout and negative work-home interference in a
nursing environment. SA Journal of Industrial Psychology.

[31] Allen TD, Herst DE, Bruck CS, Sutton M (2000). Consequences associated with work-to-family conflict: a review and agenda
for future research. J Occup Health Psychol.

[32] Lee Siew Kim J, Seow Ling C (2001). Work-family conflict of women entrepreneurs in Singapore. Women in Management Review.

[33] Byron K (2005). A meta-analytic review of work–family conflict and its
antecedents. J Vocat Behav.

[34] Hassan Z, Dollard MF, Winefield AH (2010). Work-family conflict in East vs Western countries. Cross Cultural Management: An International Journal.

